# Evaluating the effect of instruction and practice schedule on the acquisition of ECG interpretation skills

**DOI:** 10.1007/s40037-017-0365-x

**Published:** 2017-07-25

**Authors:** Sandra Monteiro, Lindsay Melvin, Joshua Manolakos, Ameen Patel, Geoffrey Norman

**Affiliations:** 1 Department of Health Research Methods, Evidence and Impact, McMaster University, Hamilton, Canada; 2 Department of Medicine, University of Toronto, Toronto, Canada; 3 Department of Medicine, McMaster University, Hamilton, Canada

**Keywords:** Distributed and massed instruction, Interleaved and blocked practice schedule, ECG interpretation, Learning

## Abstract

**Introduction:**

Evidence of the benefit of distributed instruction and interleaved practice comes from studies using simple materials (e. g. word pairs). Furthermore, there is currently no evidence of the combined impact of these strategies in undergraduate medical education. The present study evaluated the impact of varying both instruction and practice schedules for the acquisition of ECG interpretation skills.

**Methods:**

We conducted a 2 × 2 factorial study with two levels of instruction (massed and distributed) and two levels of practice (interleaved and blocked). A three-module introductory course in ECG interpretation was delivered to 80 first year medical undergraduate students. Students were assigned to one of four Instruction-Practice conditions: Massed-Interleaved, Massed-Blocked, Distributed-Interleaved and Distributed-Blocked. Learning was evaluated by a multiple choice quiz at the end of each module and a final multiple choice quiz at the end of the course.

**Results:**

End of module mean scores showed that distributed instruction was consistently superior to massed instruction (52% vs 42%, *p* < 0.01). However, there was no effect of practice and no interaction between teaching and practice methods. The delayed final test scores revealed an advantage for blocked over mixed practice (34% vs 24%, *p* < 0.05) and distributed over massed instruction (34% vs 24%, *p* < 0.05).

**Discussion:**

These results suggest that these popular strategies may have varying effects with complex learning materials. Further research is required to understand how these strategies affect the learning of simple and very complex skills.

**Electronic supplementary material:**

The online version of this article (doi: 10.1007/s40037-017-0365-x) contains supplementary material, which is available to authorized users.

## What this paper adds

Although there is evidence from experimental studies that distributing instruction over time and interleaving the sequence of practice to include items from multiple categories can have significant positive effects on learning, these strategies are not widely adopted. Importantly, the impact of both strategies has not been evaluated in medicine. This study contributes to our understanding of the impact of these strategies in a more applied education setting. Our findings suggest that interleaved practice may only become effective after sufficient mastery of the materials has occurred.

## Introduction

Medical students must master the basics of anatomy, physiology, and diagnosis over the course of 4 years of medical school. In some medical schools, such as McMaster University in Canada, this must be accomplished in a 3-year program. Electrocardiogram (ECG) interpretation, a complex skill requiring knowledge and comprehension in all three areas, is often reserved for the final year of undergraduate training. Possibly because of this limited timeframe, the standard approach to teaching is to cover new concepts in one session (typically 3 h) followed by practice problems with limited examples from one concept at a time [1]. However, there is evidence that dividing teaching into smaller sessions, sometimes called distributed instruction [1, 2], and interleaving the sequence of practice to include items from multiple categories [3] can have significant positive effects on learning [4].

Distributed instruction disperses formal teaching across several sessions that are separated by a defined period of time. This is similar to the structure of a standard undergraduate course with lectures every week or few days. Conversely, delivering instruction in one session is known as massed instruction [1]. This is similar to the academic half-day or workshop. Critically, the same content or skill is taught in one long session (massed), or taught over several short sessions (distributed). The nature of what content is distributed can be slightly different (i. e. repeat same content or smaller portions of related content) but the benefits of distributed over massed instruction are documented in several studies from various education domains [1, 2]. In medicine, performance was improved for residents following a distributed instruction schedule compared with massed instruction in Surgery [5], Urology [6] and Gastroenterology [7]. While the evidence is accumulating, the wide acceptance of formal distributed instruction in medicine has been slow [1], where formal massed instruction is characteristic of medical undergraduate curricula and postgraduate academic half-days. Thus far, no studies have examined the benefits of distributed instruction for the acquisition of ECG interpretation skills.

Interleaving different question types when working through practice problems is referred to as interleaved or mixed practice [3]; however, conventionally, instructors tend to assign ‘blocked’ practice, in much the same way as textbooks (e. g. working through t‑test problems at the end of the chapter on *t*‑tests). The superiority of interleaved practice has been demonstrated for learning in a variety of topics including, but not limited to, mathematics [8–10], music [11]; O_2_ consumption [12]; painting styles [13]; complex judgement tasks [14]; and one study on ECG interpretation [18]. The effect on ECG interpretation skills amounted to a 50% relative increase in accuracy in the interleaved practice group; however, the study only involved three ECG categories and focused only on immediate testing.

Recent studies investigating self-regulation of practice schedules showed that students prefer blocked practice schedules when left in charge of regulating their own learning [15, 16], even though the literature suggests that interleaved practice is superior [3]. One reason for this preference may be the delay in realizing benefits from interleaved practice. Magill and Hall reviewed the effects of contextual interference caused by interleaved practice that may lead to inferior performance during learning, but consistently demonstrated improved retention and transfer after delay [17]. Students may be unaware of the benefits of an interleaved practice schedule due to this delayed effect.

While there is an abundance of evidence in favour of distributed instruction and interleaved practice, only one study thus far has combined both strategies [19]. Birnbaum et al. showed that interleaved practice led to improved performance on butterfly categorization compared with blocked practice, but only for students who received massed instruction [19]. It is worthwhile to determine how instruction and practice formats affect the acquisition of more complex skills, such as ECG interpretation.

The current study examined the impact of instruction schedule and practice formats on the acquisition of ECG interpretation skills in undergraduate medical students at McMaster University. The study design is a practical trial with two levels of instruction (distributed and massed) and two levels of practice schedule (interleaved and blocked). This crossed design created four conditions: Massed-Interleaved, Massed-Blocked, Distributed-Interleaved and Distributed-Blocked. We measured performance immediately after learning, (i. e. once following instruction) and in a delayed retention test (i. e. 2–4 weeks after completion of the course).

### Research goals

The primary research goal was to extend findings that distributed instruction was beneficial to a relatively complex context of ECG interpretation. A second goal of this study was to determine if interleaved or blocked practice led to improved scores. Finally, the study evaluated whether benefits of practice or instruction schedule would be seen on an immediate test or after some delay.

## Methods

### Participants

Eighty participants were recruited from the first-year medical student class at McMaster University, in Hamilton, Ontario. McMaster University offers a 3-year medical program and students were recruited at roughly one-third of the way into the curriculum. This level of learner was selected to limit the amount of prior exposure to or instruction in ECG interpretation. In addition to the benefit of learning the advanced skill of ECG interpretation, students were offered an honorarium in the form of a gift certificate, to be received upon completion of the retention test. The students signed informed consent prior to entering the study and registered to join the training program through an online site. As students registered they were randomly assigned to one of four separate study conditions. However, once the instruction schedule was created, students were allowed to switch to a more convenient date resulting in a semi-random assignment to groups. As students were not aware of the study goals this was considered acceptable. No individual demographic data were gathered. The study was approved by the McMaster Research Ethics Board, number 11-409.

### Materials

A three-module course on the basics of ECG interpretation was delivered in a classroom with didactic instruction using PowerPoint slides projected on a screen. All students in this study were taught the same basics of ECG interpretation and introduced to the same ECG diagnoses. The three modules were delivered either all at once (massed instruction) or over 3 weeks (distributed instruction). Opportunities to practice ECG interpretation occurred within each module in either a blocked or interleaved fashion. Practice consisted of identifying two to three example ECGs of each diagnosis. All participants completed immediate testing following each module and were invited to complete a delayed test after completing the course. There were no additional learning resources used, and students were not encouraged to practice or review on their own.

Module 1 included an introductory lesson on ECG reading and introduced techniques for interpreting normal, ischaemia and pericarditis. Students were introduced to six diagnoses: (ST-elevation myocardial infarction (STEMI) in the inferior, anterior and lateral territories, ischaemia manifesting as T‑wave inversion, ischaemia manifesting as ST depression, pericarditis). At the end of module 1, students were immediately tested on an anterior STEMI, two examples of pericarditis, T‑wave inversion and normal.

Module 2 introduced narrow complex tachycardias. The module included instruction on six new diagnoses (atrial fibrillation, multifocal atrial tachycardia, atrial flutter, sinus tachycardia, atrioventricular (AV) re-entry tachycardia, AV nodal re-entry tachycardia (AVNRT)). At the end of the module 2, students were immediately tested on sinus tachycardia, multifocal atrial tachycardia, atrial fibrillation, atrial flutter and AVNRT.

Module 3 introduced bundle branch blocks and AV blocks. Students were instructed on how to interpret left bundle branch block (LBBB), right bundle branch block, first-, second- (type 1 and 2) and third-degree AV blocks. At the end of the third module students were immediately tested on third-degree AV block, first-degree AV block, LBBB, second-degree AV block type 1 and third-degree AV block.

The three learning modules were designed by three authors (LM, JM and AP). The modules were reviewed by co-authors AP, a staff internist, and JM and LM, residents in Internal Medicine, for content and to ensure an equitable level of difficulty among all modules. Two third year internal medicine residents (not the study authors) who had been accepted into a Cardiology fellowship training program were selected to be course instructors. The instructors also reviewed the modules for ease of instruction [20]. Training and practice examples of ECGs were selected from several different databases including the American College of Cardiology.

Immediate and delayed testing consisted of multiple-choice questions in a computer-based format in which students were required to select the correct diagnosis for several new ECGs. All students were administered the same tests, programmed using RunTime Revolution (version 2.8.1; Edinburgh, UK) software and presented on PC computers. Participants diagnosed five ECGs on immediate testing and 20 ECGs on delayed testing. For any one question, participants could select a diagnosis from a dropdown menu of all 19 diagnoses covered in the entire curriculum (i. e. 18 clinical diagnoses requiring treatment and the diagnosis of normal). A diagnosis could be selected more than once in a given test. Test ECGs were reviewed by authors LM, JM, and AP to ensure appropriate and similar level of difficulty to instructional ECGs provided.

### Procedure

Initially, students were assigned randomly to specific sessions: 20 students to each of the four instruction and practice conditions: Massed-Interleaved, Massed-Blocked, Distributed-Interleaved and Distributed-Blocked. Due to unavoidable scheduling changes for some students, 4 students initially assigned to the Distributed-Interleaved sessions attended the Massed-Blocked session. This resulted in uneven group numbers at the outset (see study flow diagram in the Online Supplementary Material). While we were able to track how the *N* for each group changed, we could not track individual students as they were assigned anonymous IDs once they attended the sessions.

Delayed testing occurred 2 weeks after the course ended for the distributed training groups (which is 5 weeks from the first distributed session). For the students’ convenience, two test dates were offered. Tab. 1 illustrates this schedule and the Online Supplementary Material shows how many days passed between instruction and test for all four conditions. All students were scheduled to take the same cumulative test with 20 novel ECGs. Similar to immediate testing, students selected diagnoses for each ECG from a dropdown menu of all 19 diagnoses taught in the curriculum. Students were able to complete the tests in a self-paced manner. The results of the immediate and delayed testing were only available to the study investigators. Feedback to students, in the form of correct answers, was only provided on immediate testing.

**Table 1:** Schematic depicting the time line between teaching and testing for the distributed and massed instruction groups

Week 1	Week 2	Week 3	Week 4	Week 5	Week 6	Week 7
Distributed Module 1	Distributed Module 2	Distributed Module 3	–	–	Retention Test 1 (*N* = 45)	Retention Test 2 (*N* = 6)
–	Massed 1	Massed 2	–	–	27 from Massed	3 from Massed

### Manipulation of instruction schedule

The two distributed instruction conditions required students to attend the course three times, scheduled at the same time and day of the week on consecutive weeks. The first session was 1.5 h, in order to cover the interpretation of normal ECGs as well as the remainder of the topics within module 1, a practice session and immediate test. The following week, module 2 was taught in a one-hour session, with practice followed by test. The third module was taught in a similar manner during the third week of instruction.

The two massed instruction conditions required students to attend one 3.5-hour session scheduled on a single day. The session followed the same structure as the distributed instruction conditions: module 1, 2 and 3 in that order.

### Manipulation of practice order

In the two blocked practice conditions, students practised interpreting ECGs in which diagnoses covered in the module were reviewed sequentially, grouped by diagnostic category. For example, at the end of instruction on STEMI, students saw and discussed various examples of STEMI only. The instructor then moved onto another diagnostic category (e. g. Pericarditis) and students saw and discussed examples of Pericarditis only. Emphasis was on identifying the features within each condition. No comparisons or contrasts were explicitly raised.

In the two interleaved practice conditions, students practised interpreting ECGs in which all the diagnoses covered in the module were reviewed together, comparing and contrasting features of the ECGs. The instructor first provided instruction on all the content, using a single example for each category. At the end of the instruction slides, the instructor and students reviewed an interleaved sequence of ECGs, switching between categories. For example, they would have looked at an example of an anterior STEMI, then Pericarditis, then a normal ECG and so on.

### Scoring

Learning was assessed through a computer-based multiple-choice test. Each question was assigned a score of 1 for correct or 0 for incorrect. Percent correct was calculated for each student for modules 1–3 and for the delayed test.

### Analysis

Percent correct scores of the three immediate tests were submitted to a repeated measures ANOVA with one within-subject factor of module, and two between-subject factors of instruction schedule (massed or distributed) and practice order (interleaved or blocked). Final test scores were analyzed separately using a univariate ANOVA with the same between-subject factors of instruction schedule and practice order. Alpha was set to 0.05. Additionally, a Cronbach’s alpha coefficient was calculated for the final test to determine if it was able to discriminate between students. In this context, an acceptable Cronbach’s alpha was deemed to be 0.6 or higher given the homogeneous student group. Exploratory analyses are described as necessary.

## Results

All participants indicated that they had no previous training in ECG interpretation. Eighty medical students were initially enrolled in the study and randomized to one of four conditions. However, we accommodated 4 students by allowing them to switch to a different instruction schedule after their personal schedules changed. Students were not previously aware of the nature of the conditions to avoid any selection bias so we did not correct for this. In addition, 10 students did not complete all modules and were treated as dropouts and therefore were not included in the analysis of immediate or delayed testing. As a result, the distribution of students in each condition at instruction and those included in the analysis was slightly different. The final analysis for immediate test scores included 19 students in the Massed-Interleaved condition, 24 students in Massed-Blocked, 16 students in Distributed-Interleaved and 11 students in Distributed-Blocked. A further 19 students did not complete the delayed retention test. The Online Supplementary Material shows enrolment and attrition. The mean percent correct for the four conditions on the immediate post-tests were calculated for students who attended all three modules (*n* = 70) and those who attended all modules and the retention examination (*n* = 51).

The mean percent score for the immediate tests was 38% (SD 22%; 95% CI 32–44%) on module 1, 51% (SD 22%; 95% CI 46–57%) on module 2 and 51% (SD 22%; 95% CI 46–57%) on module 3. The delayed retention test had a Cronbach’s alpha of 0.6, indicating moderate reliability and a mean percent correct of 27% (SD 15%; 95% CI 23–32%).

### Immediate tests

**Fig. 1:**
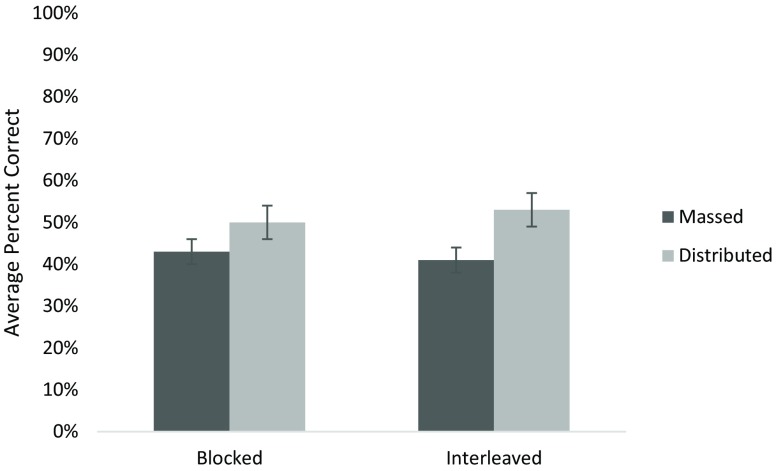
Average percent correct across all three modules (Error bars are standard error)

The repeated-measures ANOVA revealed a significant main effect of instruction schedule. As shown in Fig. 1, distributed instruction was superior to massed instruction (F (1, 66) = 7.2, *p* = 0.009). The mean percent correct for massed instruction was 42% (95% CI 38–46%) and for distributed instruction 52% (95% CI 46–57%). This benefit of distributed instruction had effect sizes ranging from a Cohen’s d of 0.2 in module 1 to 0.6 in module 3. Separate exploratory analyses of the effects of instruction and practice on module 1 (*n* = 78), 2 (*n* = 74), and 3 (*n* = 72) were also conducted using univariate ANOVA with module percent scores. There was no effect of instruction schedule on module 1 when analyzed separately in a univariate ANOVA, *p* > 0.4. Distributed instruction was superior when modules 2 and 3 were pooled in a repeated measures ANOVA (F (1.66) = 5.83, *p* = 0.02). There was a non-significant advantage for distributed instruction in module 2 (F (1.66) = 2.82, *p* = 0.1) and a significant advantage in module 3 (F (1.66) = 5.28, *p* = 0.03).

There was no main effect of practice order, with only a 1% difference in average scores between interleaved and blocked. Importantly, there were no interactions between instruction schedule and practice order.

### Retention test

**Fig. 2:**
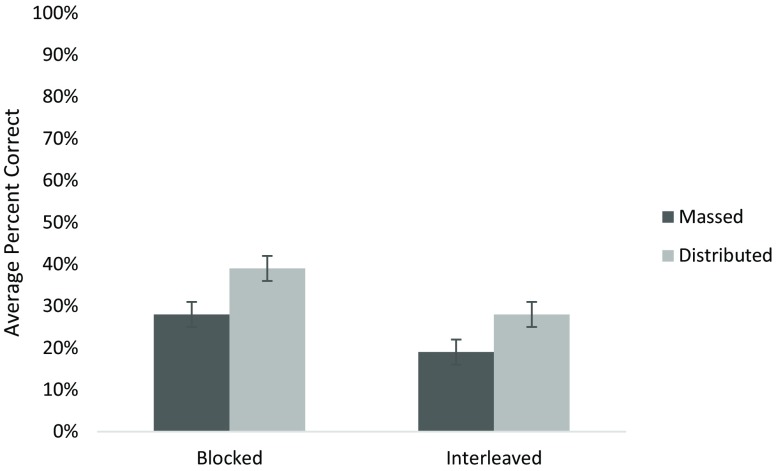
Average percent correct on the retention test for all four conditions (Error bars are standard error)

The critical issue was to determine which instruction schedule and/or practice order improved performance on a delayed cumulative retention test as shown in Fig. 2. Given the study included only very novice learners, any advantage after some time delay would be meaningful. With 51 students in the retention test and a standard deviation of 15% we had sufficient power (80%) to detect an 8% difference between groups. A univariate ANOVA on the retention test scores with two between-subject factors (instruction schedule and practice order) revealed that both factors were significant. There was an advantage for blocked practice over interleaved (F (1, 50) = 5.8, *p* = 0.02) and also for distributed instruction over massed (F (1, 50) = 5.9, *p* = 0.02) with a Cohen’s d of 0.7. The mean percent correct on the final test for blocked practice was 34% (95% CI 27–40%) and for interleaved practice 24% (95% CI 18–29%). The mean percent correct on the final test for massed instruction was 24% (18–29%) and for distributed instruction 34% (27–40%). There were no interactions between test and instruction or practice.

**Fig. 3:**
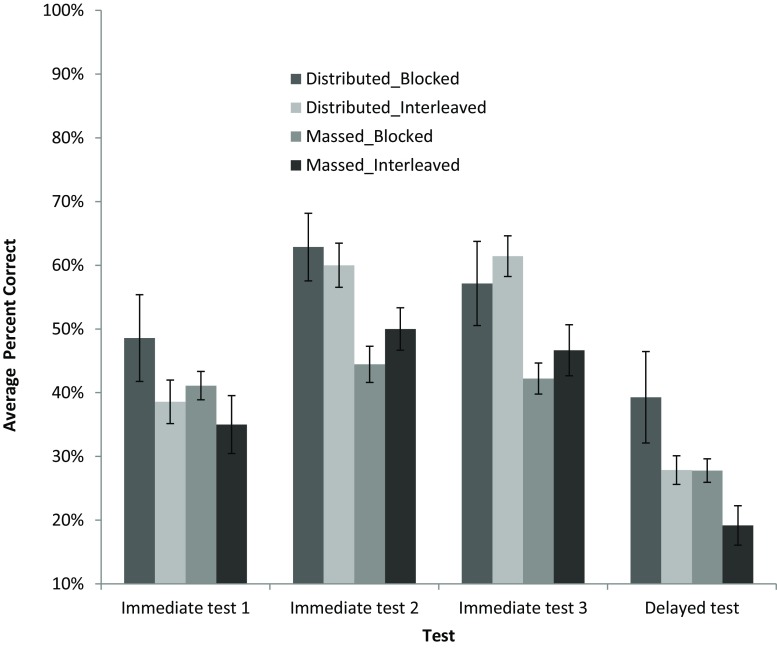
Average percent correct across all immediate tests and the delayed test. There were only five questions on all immediate tests and 20 questions on the delayed test. There is a notable increase in performance from Module 1 to 2 and a notable drop in performance after delay. Importantly, the students that retained the most were in the Distributed_Blocked group

### Effects of module content and time

Because of the variable amount of time between study and test (i. e. 3–5 weeks from module 1 to retention test) we were concerned about recency effects; there may be higher scores for questions relating to module 3, for students in the distributed instruction groups. An analysis of the retention test questions by module, however, revealed no benefits of recency on the retention test (*p* = 0.8) as all students performed equally on content: 26%, 29% and 26% on modules 1–3, respectively. Critically, there was no interaction between module content and instruction schedule (*p* > 0.6). Additionally, we conducted paired t‑tests on average module scores comparing students who dropped out before the delayed test to those who stayed and found no difference (*p* > 0.1). As performance on one test tends to be highly correlated with subsequent tests, this may be interpreted to suggest there were no differences in ability between the students who dropped out and those who stayed. Finally, to examine the potential impact of memory decay on content learned, we examined the impact of time delay on the final test score. If skill level degraded consistently over time, we would see a relationship between the length of time between instruction and final test. We conducted a univariate analysis of the final test score with number of days between instruction and test as a covariate, which revealed no relationship (*p* > 0.2). This indicated that final test scores did not show a direct relationship with time between instruction and test.

The low final test scores suggested a high degree of difficulty of the test items. Consequently, the test was administered to one of the instructors in the course, who was able to achieve a score of 85% overall, suggesting that the items did range in difficulty level.

## Discussion

### Summary of results

This study evaluated the effect of massed and distributed instruction and blocked and interleaved practice on learning ECG interpretation. The study was a practical trial examining the influence of instruction and practice strategies within a realistic education context. Intermediate medical students from an intense 3‑year medical program were recruited as naïve learners because of limited prior exposure to ECG interpretation. The learning and testing materials were authentic; validated and taught by content experts from McMaster University.

Learning was assessed both immediately following instruction and after some delay. Importantly, students in the distributed instruction group had higher scores in immediate and delayed testing. This was interpreted to suggest that there was a benefit to distributed instruction, as already reported in the literature [2, 4–7]. However, an exploratory analysis revealed that these differences were only seen in modules 2 and 3. This makes sense: distributed instruction does not have any meaning for the first module as the teaching and practice conditions for the interleaved-distributed and interleaved-massed arms were identical, and both completed the immediate test at the end of the first module.

Surprisingly and contrary to the findings of several experimental and classroom studies [8–14] there was no beneficial effect of an interleaved practice schedule. Instead there was some benefit of blocked practice, but this only appeared at the delayed retention test. This finding is not uncommon in education, indicating improved transfer over time, but limited immediate recollection [21].

### Theoretical importance

In contrast to some literature, we did not find a consistent advantage related to interleaved practice. In fact, in the retention test, blocked practice proved to be superior to interleaved practice. We hypothesize that this finding reflects the difficulty of the ECG-specific content as the average test scores were quite low in all conditions. A related interpretation of our findings is that interleaved practice may only become effective after sufficient mastery has occurred; however, this requires further study. The significance of these results should not be overlooked within the context of knowledge translation. Findings which have been replicated many times, such as the benefit of distributed instruction [1–7] or mixed practice [8–10], may not be as robust as they appear. There is growing work within applied education psychology; however, it is critical that research is taken into variable and realistic contexts. This finding also speaks to the current lack of understanding regarding the cognitive processes involved in learning under these conditions. It is still unclear what cognitive mechanisms support learning during interleaved practice and why the complexity of content may modulate the benefits of interleaving practice items. A better understanding of these mechanisms may explain the current results.

The current study demonstrated yet again that, even for very novice learners, there was an advantage to spacing instruction over time. This benefit was seen at immediate testing and after some delay, which is significant given that any difference in performance was seen with such complex skills. Critically, instruction in this study was guided and structured but with minimal feedback. Many medical education contexts encourage self-directed learning, which may not follow a distributed schedule or include any form of feedback.

Finally, although, not surprisingly, the results from the current study suggest that without formal practice between testing, there will be significant loss of skill or knowledge. Given the limited amount of time and resources available to a medical program, it is worthwhile to invest effort into improving the effectiveness of current instruction and practice opportunities for improved long-term benefits. The current results suggest that it is worth the effort to design a distributed instruction schedule for various skills; however, further work is needed to understand the impact of interleaved and blocked practice for different levels of learners and different types of skills.

### Limitations

As this study was a practical trial, it has several limitations. Firstly, there was a variation of 3–5 weeks before the retention test, with a longer delay for some students in the massed instruction conditions compared with the distributed condition (Tab. 1). This was an unfortunate consequence of scheduling challenges to find a common test date for all students, although fortunately there was an equal representation of distributed and massed students in each retention test week. While our analyses suggest these variations in delay may have had little bearing on the results, one reason to be concerned is a possible disadvantage for the massed instruction group. There are competing theories regarding the mechanisms related to loss of skill or knowledge which would predict different patterns of performance; memory decay and interference. On one hand, the concept of memory decay suggests that information is essentially lost over time [22]. If students’ skill level decayed, we might presume that the rate of loss was consistent over time, such that with longer delays from instruction to test, performance should continue to degrade following a forgetting curve [22]. This could explain the pattern of results as the students in the massed instruction group had a longer delay from teaching to test. On the other hand, the concept of interference suggests that new information might compete with old information, causing confusion over the correct response, in which case time alone would not impact performance [23]. Therefore, it is also possible that all students in our course experienced some form of interference from related knowledge, such as self-directed learning on ECGs or cardiology content; in which case the pattern of results is explained by the study manipulations of teaching schedules, and the reduced performance from instruction to final test is explained by interference. Certainly, interference could be an explanation for the lower performance of massed instruction overall as there is increased interference between content areas when they are covered all at once. Unfortunately the debate continues as to which mechanism is responsible for loss of skill or knowledge over time so we currently cannot add further interpretation [23–25].

Furthermore, the study is limited due to the relatively low performance overall reflecting the challenge of ECG interpretation for pre-clinical medical students. This limits the ability to generalize these results to other levels of trainees (such as clinically based medical students or residents) or to other tasks aside from ECG interpretation. However, this study does address the appropriate application of these strategies. Considering education more broadly, it is possible that interleaved practice will have little benefit for very novice learners in any domain. There were no attempts to objectively measure prior formal knowledge of ECGs or baseline performance in interpretation as this study was truly an evaluation of the acquisition of skill for novices. There was no expectation that students would have been able to complete a baseline assessment of ECG knowledge and all students were assumed to be of similar skill level.

Additionally, the difficulty of the material may have had an impact on students’ motivation to attend the delayed retention test. However, the dropout rate may also be attributed to students entering into different programming schedules part way through the study. Again our analyses demonstrate that dropouts most likely did not have any systematic effects on average scores.

Finally, while there are several limitations to note, these may be interpreted as a strength of a truly applied study of two popular education strategies. The current results suggest that it is not easy to simply translate strategies developed in a lab into a real medical education setting.

## Conclusion

The study showed that students in the distributed instruction group performed better overall, compared with students in the massed instruction group. In contrast to the literature on interleaved practice, we did not find a consistent advantage for interleaved practice. This finding may be explained by the hypothesis that the participants had not gained sufficient mastery to benefit from an interleaved practice order. However, future work is needed to better understand the mechanisms that support learning with varying instruction and practice formats.

## Caption Electronic Supplementary Material

Flow diagram of the study design
