# Supplementary material for: Evaluating the effect of instruction and practice schedule on the acquisition of ECG interpretation skills
Source: Perspect Med Educ. 2017 Jul 25;6(4):237–45. doi: 10.1007/s40037-017-0365-x (PMC5542896; doi:10.1007/s40037-017-0365-x)
Supplement: Supplementary file 1 — Flow diagram of the study design [file 40037_2017_365_MOESM1_ESM.docx]

Retention test

(n = 51)

May 22 to June 4

FINAL DELAYED TEST

ANALYSIS OF IMMEDIATE QUIZZES

19 additional participants lost at retention test to attrition

Distributed

Blocked

(n = 11)

Massed

Interleaved

(n = 19)

Distributed

Interleaved

(n = 16)

Distributed

Blocked

(n = 20)

April 19, 26 and May 3

10 participants excluded from analysis of immediate testing for not completing all 3 modules

Unexpected change in training schedules resulted in migration of some students to other conditions

Massed

Blocked

(n = 24)

Massed

Blocked

(n = 24)

April 29

INSTRUCTION/PRACTICE

CONDITION

Massed

Interleaved

(n = 20)

April 22

Distributed

Interleaved

(n = 16)

April 17, 24 and May 1

ENROLLMENT

Randomized

20 per Condition

80 McMaster 1^st^ year medical students

80 students enrolled
